# Differences in clinical variables of cervical cancer in women with schizophrenia

**DOI:** 10.1192/j.eurpsy.2024.660

**Published:** 2024-08-27

**Authors:** F. Casanovas, F. Dinamarca, S. Oller, A. Trabsa, L. Martínez-Sadurní, R. Rodríguez-Seoane, N. Zabaleta, L. M. Martin, V. Perez-Sola, A. I. Ruiz

**Affiliations:** ^1^Institut de Salut Mental, Hospital del Mar, Barcelona; ^2^Centro de Investigación Biomédica en Red de Salud Mental (CIBERSAM), Madrid; ^3^Departmeny of Psychiatry, Hospital de la Santa Creu i Sant Pau; ^4^Programa de doctorat en psiquiatria i medicina legal, Universitat Autònoma de Barcelona, Barcelona, Spain

## Abstract

**Introduction:**

Schizophrenia is associated with a reduced life expectancy, not only because of suicide, but also medical causes such as cancer. Standardized mortality for cancer is higher in patients with schizophrenia, specially for lung, breast and colorectal locations (Ni et al, 2019). Other less frequent tumor locations have not been deeply studied.

Thir mortality gap could be related to a delayed diagnosis due to several reasons, such as lower inclusion in screening programs (Solmi et al, 2019). Since cervical cancer has a very efficient screening technique, women with schizophrenia and cervical cancer could have a worse prognosis because of a delayed diagnosis. However, there is a lack of research in this tumor location.

**Objectives:**

To analyze clinical differences in women with cervical cancer with and without a diagnosis of schizophrenia.

**Methods:**

We carried out a retrospective cohort analysis with adult patients from the cancer registry of Hospital del Mar diagnosed between 1997 and 2021. The information was crossed with the Minimum Basic Data Set (MBDS) to identify those cancer patients with a diagnosis of schizophrenia using International Classification of Diseases (ICD) 9 codes 295*. The sociodemographic variables were age and sex. The clinical oncological variables included tumor location, place of first conultation, stage, first treatment intention, vital status and place of decease. We used t-student for continuous data and Chi-squared test for categorical variables. We performed a post-hoc analysis using Bonferroni correction for multiple comparisons to identify specifically which categories were significantly different between groups.

**Results:**

We identified 13 women with schizophrenia and cervical cancer, and 1354 women with cervical cancer without schizophrenia. The proportion of this location was higher in the schizophrenia group (8% of all cancers vs. 4.4%; p=0.03). The proportion of diagnoses through screening programm was significantly lower (7.7% vs 14.6%; p=0.04). There was a trend of fewer diagnoses in situ in patients with schizophrenia (30.8% vs 55.6%) and less radical intention as first treatment option (15.4% vs 3.5%) but without statistical significance in both cases. There was a higher proportion of deceased patients in the group with schizophrenia (46.2% vs 15% p=0.002), and also a higher proportion of deaths outside hospital facilities (30.8% vs 6.6%; p=0.003).

**Image:**

**Figure:**
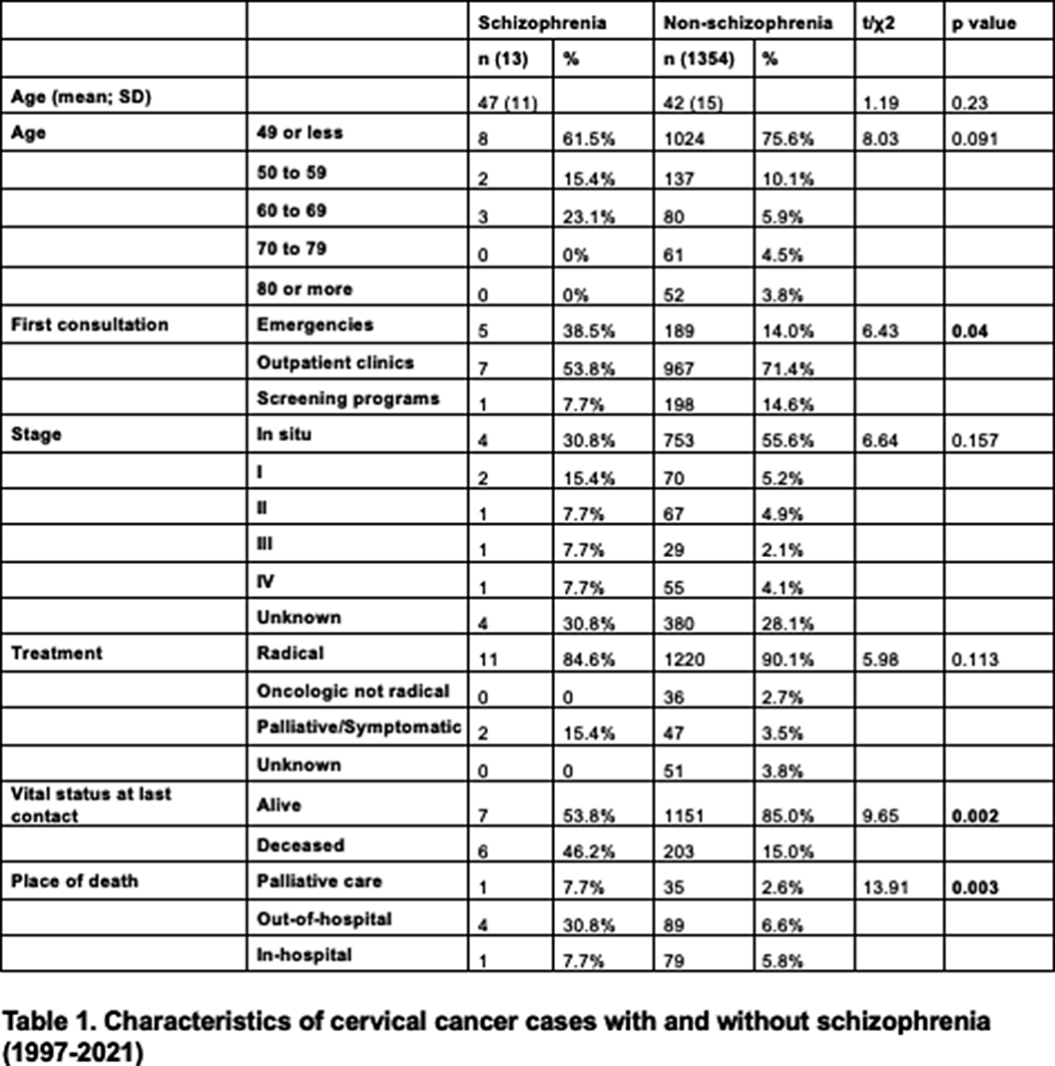

**Conclusions:**

Women with schizophrenia receive less diagnoses of cervical cancer through screening programs and more in emergency facilities, which could lead to more advanced stages and fewer indication of radical treatments. This ultimately leads to a higher proportion of deaths, and more frequently outside of hospital facilities.

Our data supports the idea that the increased mortality for cancer is related to a delayed diagnosis. Women with schizophrenia need special care to ensure their inclusion in early detection programs for cancer.

**Disclosure of Interest:**

None Declared

